# A Non-Matrix-Matched Calibration Method for In Situ Major and Trace Element Analysis of Scheelite by Nanosecond LA-ICP-MS

**DOI:** 10.3390/molecules29010051

**Published:** 2023-12-20

**Authors:** Xijuan Tan, Honghao Tian, Lin Lu, Dongyang Xiong, Ting Liang

**Affiliations:** 1 Laboratory of Mineralization and Dynamics, College of Earth Sciences and Land Resources, Chang’an University, 126 Yanta Road, Xi’an 710054, China; honghao1221@163.com (H.T.); xdyran08@163.com (D.X.); liangt@chd.edu.cn (T.L.); 2 Shaanxi Mineral Resources and Geological Survey, Shaanxi Institute of Geological Survey, Xi’an 710068, China; lulin995280@126.com

**Keywords:** major and trace element, scheelite sample, non-matrix-matched calibration, nanosecond LA-ICP-MS

## Abstract

In this work, a reliable and robust in situ non-matrix-matched calibration method is proposed for element composition determination in scheelite samples. With external calibration against the silicate glass standard reference material NIST SRM 610, the concentrations of both major elements (Ca and W) and trace elements (Si, Fe, Mo, Y, rare earth elements, etc.) in scheelite are determined using an ArF 193 nm excimer nanosecond laser ablation-inductively coupled plasma mass spectrometer (LA-ICP-MS). Here, the ablation was performed by hole drilling under a helium (He) environment using a laser spot size of 35 μm and a laser repetition of 5 Hz, and the aerosols were then transported to a quadrupole ICP-MS by a mixture of He and make-up gas argon (Ar) with a total gas flow rate of 1.6 L/min. Results showed that there was no apparent matrix effect between the NIST SRM 610 and scheelite by this proposed method. With internal standardization against W, the obtained concentrations of CaO and WO_3_ were found to yield an average matrix CaO/WO_3_ mass fraction ratio of 0.245 (2σ = 0.003, *n* = 19), which agreed well with the value of 0.243 (2σ = 0.002, *n* = 15) from electron probe microanalysis (EPMA). Furthermore, the accuracy of trace element analyses with this proposed non-matrix-matched calibration in situ method was evaluated by comparing the concentration results with those from bulk analysis by solution nebulizer ICP-MS (SN-ICP-MS). It was found that the quantification results from LA-ICP-MS and SN-ICP-MS were comparable, in particular showing a relative concentration bias of the total ∑REE+Y contents of less than 2%. This confirmed that scheelites can be accurately analyzed in situ by LA-ICP-MS without matrix-matched calibration standards. By using this developed in situ method, the element compositions in a series of scheelite samples from different W-associated deposits in China were successfully quantified, promising further genetic process investigation and associated geologic activities of the polymetallic resources.

## 1. Introduction

Scheelite, which has a chemical composition of CaWO_4_ with a simple crystal structure of tetrahedral [WO4]^2−^ groups and irregular dodecahedral [WO8]^4−^ groups [1], is a widespread accessory mineral in hydrothermal associated mineral deposits, such as tungsten deposits, gold deposits, and Sn and Mo deposits [2,3,4]. Due to the similarities of ironic radii and the tetragonal symmetry crystal structure of Ca-bearing minerals, a wide range of elements involving Sr, Y, Mo, Pb, and rare earth elements (REEs) are easily incorporated into scheelite via the substitution of Ca^2+^ or W^6+^ in the crystal lattice [5]. This makes scheelite a well-known host of various trace elements, generally exhibiting distinct REE patterns and trace element characteristics [6,7].

During the last several decades, scheelite has been extensively utilized in the investigation of deposit types, metallogenic settings, ore genesis, physicochemical conditions, and the evolutionary history of the mineralizing fluids via its trace element compositions [8,9,10,11]. By analyzing the REE patterns and trace element systematics of scheelite samples, Ghaderi et al. [12] discussed the rock types and physical conditions in the Kalgoorlie-Norsman region of West Australia at the time of hydrothermal activity. Song et al. [13] applied the element geochemistry of scheelite samples to source identification and oxide/redox condition prediction of the ore-forming fluids for skarn-type W-Mo deposits in east China. Poulin et al. [14] assessed the capability of scheelite as a good ore-deposit discriminator via the comprehensive study of its trace element and REE chemistry. Li et al. [15] tracked the dynamic hydrothermal processes through REE fractionation patterns and Sr-Nd isotopes of scheelite samples from the Yangjiashan W deposit in south China. With trace element and Sr isotope geochemistry of skarn-hosted scheelite samples, Dai et al. [16] constrained the source and evolution of the ore-forming fluid of the Cuonadong Sn-W-Be polymetallic deposit in south Tibet of China. 

The trace elements of scheelites were conventionally analyzed by inductively coupled plasma atomic emission spectroscopy (ICP-AES) [17] or solution nebulizer ICP mass spectrometry (SN-ICP-MS) [18,19]. By using such techniques, sample decomposition is mandatory prior to the quantification of scheelite trace elements. Generally, scheelite is digested through either alkali fusion/sintering followed by acid leaching [18,20,21] or acid decomposition with low-temperature attack [22] and/or the Teflon bomb method [17,19]. These methods, however, suffer from the time-consuming sample preparation process, the relatively high content of REEs from fusion reagents, and the risk of element loss during digestion procedures. Laser ablation ICP-MS (LA-ICP-MS), an in situ technique [23] for micro-scale major and trace element analysis in solid samples [24,25], has been extensively employed to quantify element composition in scheelite using a non-matrix-matched standard calibration method. Sylvester et al. [26] first reported the usage of LA-ICP-MS in quantitative element composition analyses of scheelite, giving W measurements that fell within 5% of the concentrations expected from electron probe microanalysis (EPMA). However, they found that W progressively fractionated from Ca in the silicate glass standard reference material NIST SRM 610 but not in the scheelite during ablation. The authors thus recommended the data reduction should be performed using the early maximum count rates for W and Ca rather than the mean count rates of the whole ablation. Fu et al. [27] concluded that the matrix effect between NIST SRM 610 and scheelite can be circumvented with the usage of Ca as the internal standard element and a 193 nm excimer laser operated at a low laser repetition rate of 5 Hz. However, Hao et al. [28] argued that the matrix effect was negligible when the ablation was conducted using a 193 nm excimer laser at a 7 Hz repetition rate and 44 μm spot size. Recently, Xiao et al. [29] studied the ablation behaviors of scheelite and NIST SRM 610 with a spot size of 10~90 μm, revealing that a spot size of ≥32 μm was preferred for scheelite regardless of pulse number, while the favored spot size for NIST SRM 610 was highly dependent on the utilized pulse number. Collectively, there has been no consistent approach for the element composition in scheelite by LA-ICP-MS using NIST SRM 610 as the external calibration standard. 

In this work, a non-matrix-matched calibration method against NIST SRM 610 for accurate major and trace element determination in scheelite samples by using nanosecond LA-ICP-MS was proposed. Here, the ablation behaviors of elements W and Ca in scheelite were studied in detail. Additionally, the matrix effect and quantification accuracy of this in situ method were evaluated. Finally, this developed LA-ICP-MS approach with optimized configuration was employed to determine the element compositions in a series of scheelite samples which were collected from different W-associated deposits in China.

## 2. Results and Discussion

### 2.1. Element Compositions of Scheelite Samples by EPMA Analysis

By using EPMA, the concentration levels of matrix components Ca and W together with elements Si, Na, Mg, Ca, Mn, Fe, and Mo in the studied scheelite samples were obtained. The results are summarized in Table 1, showing that the contents of Na, Mg, Mn, Fe, and Mo were nearly within the detection limits. It was also found that the average concentrations of SiO_2_ ranged from 0.14 to 0.21%, and the relative standard deviations (RSDs) were higher than 15% (*n* = 3). Since the quantification was carried out on the region that appeared free of surface-near inclusions or cracks by backscattering electron image inspection, we assumed that the large RSDs of SiO_2_ contents might be due to heterogeneities or inclusions hidden under the sample surface. The concentration values of CaO and WO_3_ were observed to be within 18.74–19.83% and 78.28–80.87%, respectively, with RSDs of less than 2.83% (*n* = 3). The corresponding matrix CaO/WO_3_ mass fraction ratios for these samples were found in the range of 0.235 to 0.250 and yielded an average value of 0.243 (2σ = 0.002, *n* = 15).

### 2.2. Ablation Behaviors of Ca and W in LA-ICP-MS Analysis

**Figure 1:**
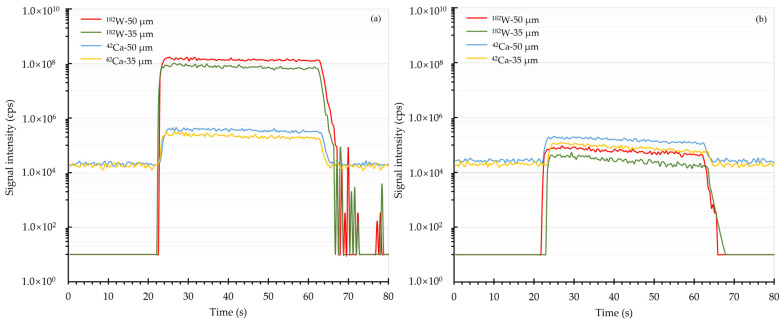
Transient signal profiles of Ca and W. Panel (**a**) and panel (**b**) were the transient signal results of scheelite and NIST SRM 610, respectively. Here 50 and 35 μm spot sizes were utilized.

The ablation behaviors of Ca and W in scheelite and NIST SRM 610 were investigated by monitoring the transient signals over the course of one single spot analysis. With a fixed energy fluence of 2.51 J/cm^2^, the transient signal profiles of Ca and W using 50 and 35 µm spot sizes were graphically shown in Figure 1. It was observed that the intensities of both Ca and W in scheelite and NIST SRM 610 were relatively steady during the whole ablation period. Furthermore, the element fractionation of W was then estimated in terms of the ratios of integrated signals of W, which were normalized to the integrated signals of Ca, from the second 20 s to that from the first 20 s [30]. The element fractionation analyses showed that the fractionation factor of W in scheelite for one continuous ablation was 1.07 when using a 50 µm spot size, while this value declined to 1.01 when using a spot size of 35 µm. It is thus clear that a smaller spot size of 35 µm produces less element fractionation effect on scheelite sample analysis. When it comes to NIST SRM 610, the fractionation factors were found to be 1.01 using a 50 µm spot size and 1.02 using a 35 µm spot size, showing that there was no significant influence from the utilized spot size. Apparently, the reported phenomenon that W progressively fractionated from Ca in NIST SRM 610 [26] did not occur under this current instrument configuration.

**Figure 2:**
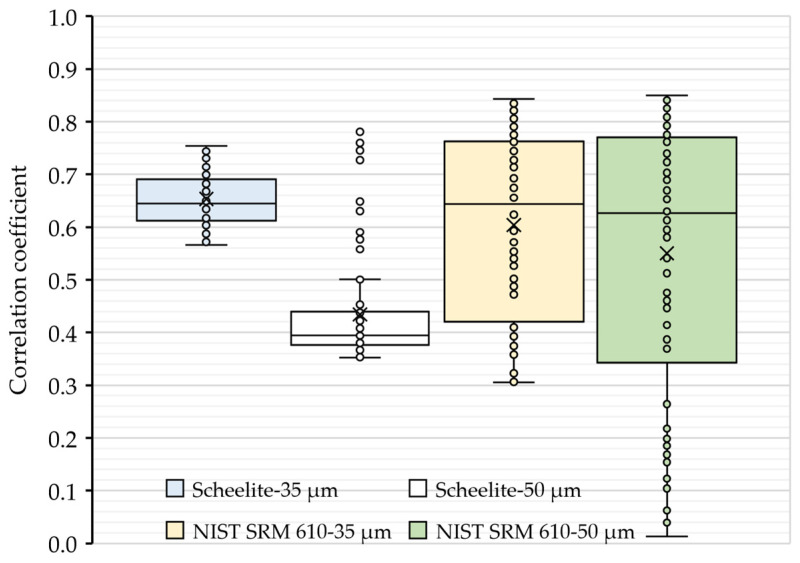
Correlation coefficients of the signal intensity of Ca with W in an ablation. The correlation coefficients were obtained from the intensity values of Ca and W collected at each sweep of the ICP-MS detector after the ablation started. Boxes in blue and white are results for scheelites, while boxes in light gold and green are results for NIST SRM 610.

To further evaluate the ablation behaviors, the correlation coefficients of transient signal intensities between Ca and W within the ablation process at 35 and 50 µm spot sizes were analyzed (see Figure 2). As shown in Figure 2, the values of the correlation coefficient were found to be less than 0.9, revealing that the two elements had a relatively independent relationship during the interactions of the target (i.e., scheelite and NIST SRM 610) with pulse lasers. However, it is worth noting that there were outliers of the correlation coefficients in the ablation of scheelite using a 50 µm spot size, which was consistent with the slightly higher element fractionation effect of W in scheelite samples. Hence, a 35 µm spot size was used as default in the subsequential quantification analyses.

### 2.3. Matrix Effect and Quantification Accuracy of Major and Minor Elements Using LA-ICP-MS

With NIST SRM 610 as the calibration standard, the five scheelite samples were quantified by LA-ICP-MS using a hole drilling strategy under 5 Hz laser repetition, 35 µm spot size, and 2.51 J/cm^2^ of energy fluence. Results showed that the concentrations of CaO were within 19.03–20.16% (RSDs < 2.86%, *n* ≥ 3), and WO_3_ was found at levels between 79.43% and 80.72% (RSDs < 0.74%, *n* ≥ 3). It is clear that the content levels of CaO and WO_3_ were highly in agreement with the pure stoichiometric scheelite (19.48% of CaO and 80.52% of WO_3_), which confirmed the nearly ideal stoichiometric composition of the studied scheelite samples. The yielded average matrix CaO/WO_3_ mass fraction ratio of 0.245 (2σ = 0.003, *n* = 19) also showed consistency with that obtained by EPMA. Hence, the matrix effect from the non-matrix-matched standard glass NIST SRM 610 can be neglected for scheelite quantification when using the proposed LA-ICP-MS method. 

**Figure 3:**
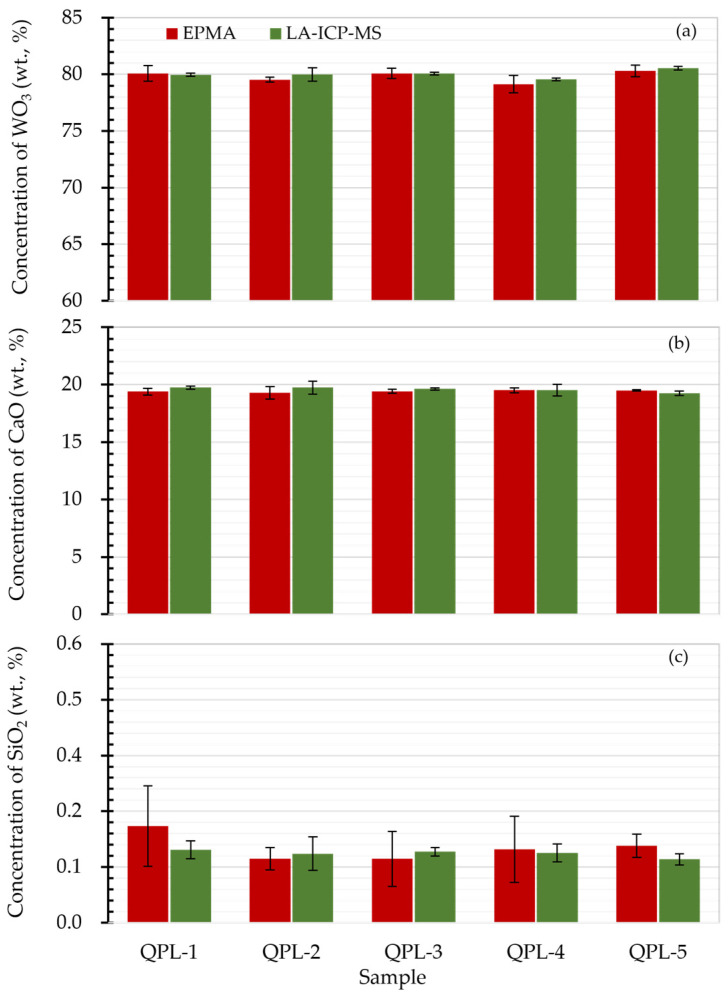
Result comparison of scheelite determined by EPMA and LA-ICP-MS. Panels (**a**–**c**) were the concentration comparison for WO_3_, CaO and SiO_2_ in scheelite samples from EPMA and LA-ICP-MS analyses. Here the error bar was the standard deviation for repetitive quantifications. The LA-ICP-MS analysis was performed using 5 Hz laser repetition, 35 μm spot size, and 2.51 J/cm^2^.

To further assess the quantification accuracy of scheelite samples by the proposed LA-ICP-MS, concentration results of CaO, WO_3_, and SiO_2_ were then compared to the values obtained by EPMA. As shown in Figure 3a,b, it is clear that the concentrations of matrix CaO and WO_3_ from LA-ICP-MS analysis agreed well with results from EPMA analysis, giving concentration ratios within 0.98–1.01 (see Figure S1). Additionally, the minor component SiO_2_ with values of 0.13–0.19% from LA-ICP-MS analysis was found to fall within the concentration range given by EPMA (see Figure 3c). It is worth noting that the concentration results of SiO_2_ from LA-ICP-MS analysis exhibited better precision with standard deviations of less than 0.04%, which might be attributed to the larger sample mass sampled via laser ablation and, thus, the micro-heterogeneities or inclusions hidden under the sample surface such as QPL-1 were not noticeable.

### 2.4. Quantification Accuracy of Trace Elements Using LA-ICP-MS and SN-ICP-MS

Apart from the composition quantification of major and minor elements, the analytical capability of this proposed in situ method for trace elements in scheelite was also evaluated. With concentration values from bulk solution analysis using SN-ICP-MS as the reference, the concentration ratios are shown in Figure S2, and the concentration results of selected trace elements Y and REEs are summarized in Table 2. It is clear from Figure S2 and Table 2 that there were no significant differences in the results for the studied scheelite samples from LA-ICP-MS analyses to those from solution-based SN-ICP-MS measurements, which gave concentration ratios in the range of 0.8 to 1.2, in particular showing a relative concentration bias of the total ∑REE+Y contents of less than 2%. This demonstrated the robustness of this developed LA-ICP-MS approach with NIST SRM 610 as the calibration standard for trace element quantification in scheelite samples. On the other hand, the comparable content levels from the in situ and bulk solution analyses revealed a relatively homogeneous distribution of trace elements in the studied scheelite samples.

### 2.5. Application to Scheelite Sample Quantification by LA-ICP-MS

Based on the results discussed above, the proposed LA-ICP-MS method was then applied to element quantification of a suite of scheelite samples from different W-associated deposits in China. Here, samples QPL-6~QPL-10 are also from the Qipangou deposit of Shaanxi, samples WTG-1~WTG-10 are from the Wutonggou deposit of Xinjiang [31], and samples XDS-1~XDS-10 are from Xiaodushan deposit of Gansu [32]. The quantification results are compiled in Table S1.

It is clear from Table S1 that the matrix components in the scheelite samples were in a range of 18.64–19.89% for CaO and 79.76–80.95% for WO_3_, from which the regional differences among the three deposits could not be distinguished. However, the three deposits were found to differentiate in terms of trace elements. Scheelite samples from the Qipangou deposit showed the lowest content level of Mg but the highest concentrations of Si, Fe, and Sr, with Na, Mn, Ga, Th, and U below the detection limits of the used instrument. Samples from the Xiaodushan deposit were observed to enrich elements Na, Mg, Mn, Nb, Pb, Y, and REEs, and contained total ∑REE+Y contents up to thousands of μg/g, which were distinctly higher than those from the other two deposits. Furthermore, elements Th and U were detectable only in the scheelite samples from the Xiaodushan deposit, showing contents of 0.19–0.50 and 0.05–0.25 μg/g, respectively. For the Wutonggou deposit, the scheelite samples enriched element Mo in particular, showing concentrations over a wide range of 259.7–924.6 μg/g except for sample WTG-7, which contained 17.08 μg/g of Mo. It is also worth noting that there were four levels of the sum concentrations of Y and REEs in scheelite samples from the Wutonggou deposit, and the total ∑REE+Y contents generally followed an increasing trend of around 1.82, 11.08–31.16, 74.46–128.7, and about 238.7 μg/g. This might be due to the different evolution stages or fluid activities during scheelite mineralization in the Wutonggou deposit. However, the aim of this study was not specifically to investigate the scheelite mineralization process. Collectively, one can note that the characteristics of trace elements in these scheelite samples can provide valuable information in W-associated deposit fingerprinting.

## 3. Materials and Methods

### 3.1. Instrumentation and Operating Conditions

The measurements were carried out on an Agilent 7700x ICP-MS (Agilent, Santa Clara, CA, USA), connected to a 193 nm ArF excimer nanosecond LA system. This Analyte Excite LA system (Photon Machines, Thousand Oaks, CA, USA) provides a maximum energy fluence of 15 J/cm^2^ and contains a HelEx Active two-volume LA cell [33]. To enhance isotope signal sensitivity, the torch of the utilized ICP-MS was equipped with a Pt shielding plate and a silicon shielding cap. Generally, the signal intensity for 1 μg/g of U can reach 2500 cps/s when using a 5 Hz laser repetition rate and a 40 μm spot size. A “cylinder shape signal stabilizer” with a volume of 21.2 mL [34] was installed after the LA cell to reduce the signal oscillations during the measurements. Here, ablation occurred in a helium atmosphere (He, 99.999% purity). Argon gas (Ar, 99.996% purity) was used as the make-up gas and mixed with the sample aerosol from the ablation cell by a T-connector before entering the ICP.

The LA-ICP-MS was optimized daily to achieve the highest possible sensitivity for low- to high-mass isotopes before scheelite analyses. After the system had been stabilizing for at least one hour, the carrier gas flow rates of He were optimized through ablating NIST SRM 610 silicate glass using a fixed spot size of 40 μm and a repetition rate of 5 Hz. Apart from analyte sensitivity, the daily optimization included the oxide formation control with a ThO^+^/Th^+^ ratio lower than 0.5% and an achieved ^238^U^+^/^232^Th^+^ signal intensity ratio near 1.05. Other parameters (including sampling depth and lens voltages) and the ICP-MS instrument’s P/A factor calibrations were updated on a daily basis. Typical operating parameters for the ICP-MS and LA system are summarized in Table 3.

### 3.2. Sample Description and Handling

Five scheelite samples from the W-associated mining deposit in Shaanxi Province, China [35] (Qipangou deposit: QPL-1, QPL-2, QPL-3, QPL-4 and QPL-5) were selected in this method of investigation work.

All scheelite samples were prepared in the form of thin sections, for which the scheelite samples with a standard thickness of 30 μm were carefully mounted on glass beads. When the surfaces of all the samples were polished, the samples were cleaned using ethanol (99.7%) followed by sonication in ultrapure water (Milli-Q, Millipore, Bedford, MA, USA). The NIST SRM 610 reference glass was used for calibration and the preferred values from the GeoReM database [36] were used as references. Before LA sampling, the surfaces of all the standard material and samples were carefully cleaned using ethanol (99.7%) and let dry.

### 3.3. Data Acquisition and Processing

Each LA-ICPMS analysis consisted of a 20 s background signal acquisition and 40 s data collection while ablating standards and the samples. One complete assay cycle consisted of a sequence of 2 spot analyses of NIST SRM 610, 6 to 10 spot analyses of scheelite samples, and 2 spot analyses of NIST SRM 610.

All data were collected in a mode of time-resolved analysis and data reduction was carried out using “Stalquant”, an in-house data reduction software developed by the Günther group in ETH [37]. Here, NIST SRM 610 was defined as the external standard. Quantification was performed using W as the internal standard element or via matrix normalization (100 wt % oxides) [38]. In this work, the major element concentration results of scheelite samples were expressed as 100 wt % oxide, while the trace element concentrations were given as μg/g.

### 3.4. EMPA

EPMA analyses were performed using a JEOL JXA-8100 instrument (Jeol Ltd., Tokyo, Japan), with apparatus details given in our previous work [39]. In this current work, all data for the scheelite samples were obtained using an acceleration voltage of 15.0 kV, a probe current of 20 nA, and an electron beam diameter of 1 µm. Acquisition times were 20 s on peak and 10 s on each background position. For all elements, the K_α_ lines were used, except for Mo, where the L_α_ line was measured. Here, Si, Na, and Mg were measured on TAP; Ca, Mo, and W were analyzed on PET, Fe; and Mn values were determined on LIF. The natural minerals wollastonite, albite, forsterite, fayalite, and pyrophanite were used as standards for Si, Na, Mg, Fe, and Mn, respectively. Scheelite was the standard for Ca and W, and a pure metal was used for Mo. At least 3 points were measured for each sample and the element concentrations were calculated using the CitZAF interelement correction model [40]. The results were expressed as oxide form in wt %.

### 3.5. SN-ICP-MS

The solution analyses of trace elements in scheelite samples were finished by a ThermoFisher Scientific X7 ICP-MS (Waltham, MA, USA). The scheelite samples were digested using an in-house high-pressure closed acidic decomposition method [41] with small modifications. With the addition of 0.5 mL of HF and 1.0 mL of HNO_3_, a sample of 50 ± 0.5 mg in the Teflon bombs was evaporated to incipient dryness at 140 °C. Then, 0.5 mL of HF, 0.5 mL of HCl, and 1.0 mL of HNO_3_ were added to the sample. Thereafter, the bomb was tightly closed and put into an oven at 185 °C for 48 h. After cooling, the sample was evaporated to incipient dryness at 140 °C, and the residue was re-dissolved and dried twice using 1.0 mL of HNO_3_. With 2.5 mL of 40% HNO_3_ (*v*/*v*) added, the sample solution was heated at 135 °C for 6 h with bombs sealed and then aged overnight. Then the sample solution was transferred to a PET bottle and gravimetrically diluted to 50 ± 0.5 g using 2% HNO_3_ (*v*/*v*) solution. After aging overnight, the sample was directly taken for quantification of trace elements by ICP-MS.

## 4. Conclusions

In this work, a reliable and accurate LA-ICP-MS method using NIST SRM 610 as an external calibration standard for major and trace element quantification of scheelite samples was proposed. The ablation behavior study showed that less element fractionation occurred for scheelite analyses when using a smaller laser spot size, while the element fractionation was negligible for NIST SRM 610 analyses irrespective of the utilized spot size. With a laser spot size of 35 μm and a laser repetition of 5 Hz, no apparent matrix effect was observed between the NIST SRM 610 and scheelite using this current instrumental configuration. The obtained concentrations of matrix components CaO and WO_3_ were highly consistent with those from EPMA analysis. Furthermore, the concentration results of trace elements of the studied scheelite samples by this proposed in situ method were comparable with those from bulk solution analysis by SN-ICP-MS, in particular showing a relative concentration bias of the total ∑REE+Y contents less than 2%. This developed method was then applied to element composition determination in a suite of scheelite samples from different W-associated deposits in China. Despite there being no significant concentration differences of the matrix components, the regional deposits can be distinguished by trace elements, which was valuable for further investigation of the genetic process and associated geologic activities of the polymetallic resources.

## Figures and Tables

**Table 1:** Chemical compositions of scheelite samples by EPMA ^1^.

Sample		CaO	WO_3_	SiO_2_	Na_2_O	MgO	MnO	FeO	MoO_3_
QPL-1	QPL-1_1	19.08	80.87	0.30	0.019	LD	LD	0.002	0.025
	QPL-1_2	19.60	79.78	0.19	LD	LD	LD	0.022	LD
	QPL-1_3	19.54	79.64	0.14	0.007	0.004	LD	0.013	LD
	Average	19.40	80.09	0.21	0.009	0.001	LD	0.012	0.008
	2σ	0.33	0.78	0.10	0.011	/	/	0.012	/
QPL-2	QPL-2_1	18.74	79.77	0.11	LD	0.021	LD	0.021	0.102
	QPL-2_2	19.35	79.48	0.16	LD	0.034	LD	LD	0.068
	QPL-2_3	19.83	79.35	0.14	0.036	0.001	LD	LD	LD
	Average	19.31	79.54	0.14	0.012	0.019	LD	0.007	0.057
	2σ	0.63	0.25	0.03	/	0.019	/	0.014	0.060
QPL-3	QPL-3_1	19.25	80.61	0.10	LD	LD	0.029	0.038	0.008
	QPL-3_2	19.61	79.87	0.21	0.058	LD	LD	LD	LD
	QPL-3_3	19.39	79.79	0.11	LD	0.017	0.011	LD	0.034
	Average	19.42	80.09	0.14	0.019	0.006	0.013	0.013	0.014
	2σ	0.21	0.52	0.07	/	/	0.017	/	0.021
QPL-4	QPL-4_1	19.68	79.37	0.14	0.074	0.033	0.014	0.038	LD
	QPL-4_2	19.59	78.28	0.24	0.186	0.036	0.021	0.054	0.017
	QPL-4_3	19.27	79.78	0.10	LD	0.020	0.015	LD	0.042
	Average	19.52	79.14	0.16	0.130	0.030	0.017	0.031	0.020
	2σ	0.25	0.90	0.08	0.091	0.010	0.004	0.032	0.024
QPL-5	QPL-5_1	19.45	80.36	0.17	0.045	0.011	0.011	0.014	dl
	QPL-5_2	19.57	80.79	0.14	LD	0.034	LD	0.009	0.017
	QPL-5_3	19.50	79.75	0.19	0.071	LD	0.019	LD	LD
	Average	19.51	80.30	0.17	0.039	0.015	0.010	0.008	0.006
	2σ	0.07	0.60	0.03	0.041	0.020	0.011	0.008	/

^1^ LD denotes below detection limits.

**Table 2:** Content results of Y and REEs in scheelite by SN-ICP-MS and LA-ICP-MS ^1^.

Element	QPL-1				QPL-2				QPL-3				QPL-4				QPL-5			
	SN-ICP-MS		LA-ICP-MS		SN-ICP-MS		LA-ICP-MS		SN-ICP-MS		LA-ICP-MS		SN-ICP-MS		LA-ICP-MS		SN-ICP-MS		LA-ICP-MS	
	Content μg/g	2σ	Content μg/g	2σ	Content μg/g	2σ	Content μg/g	2σ	Content μg/g	2σ	Content μg/g	2σ	Content μg/g	2σ	Content μg/g	2σ	Content μg/g	2σ	Content μg/g	2σ
Y	35.70	1.39	33.42	1.17	38.92	1.21	39.46	1.40	35.50	1.1	33.79	0.37	21.20	0.66	19.73	0.19	24.60	0.83	24.74	0.70
La	3.04	0.21	3.43	0.21	2.56	0.6	2.69	0.17	2.46	0.12	2.32	0.31	1.47	0.07	1.48	0.02	1.83	0.09	1.74	0.16
Ce	13.01	0.64	13.74	0.30	12.01	0.56	11.54	0.45	10.10	0.19	10.24	0.29	6.08	0.09	6.37	0.04	8.44	0.13	8.49	0.24
Pr	2.56	0.19	2.77	0.05	2.06	0.09	2.06	0.06	1.84	0.06	1.98	0.02	1.18	0.04	1.35	0.02	1.52	0.05	1.53	0.04
Nd	15.30	0.71	15.97	0.54	12.61	0.50	12.26	0.38	10.80	0.27	11.48	0.13	7.11	0.18	7.46	0.10	9.06	0.23	8.90	0.11
Sm	5.29	0.36	5.71	0.28	4.60	0.31	4.75	0.50	3.86	0.15	4.40	0.05	2.53	0.10	2.37	0.04	3.18	0.13	3.20	0.09
Eu	2.77	0.17	2.60	0.53	1.66	0.08	1.61	0.06	2.41	0.05	2.02	0.02	0.93	0.03	1.06	0.05	1.13	0.04	1.14	0.03
Gd	5.87	0.36	6.17	0.81	5.28	0.17	5.12	0.17	4.50	0.08	4.31	0.07	2.89	0.05	3.08	0.04	3.62	0.07	3.58	0.15
Tb	1.04	0.08	0.96	0.27	0.94	0.03	0.91	0.03	0.80	0.02	0.84	0.01	0.52	0.02	0.51	0.01	0.65	0.02	0.65	0.02
Dy	5.87	0.34	5.90	0.77	5.36	0.16	5.20	0.17	4.57	0.11	4.44	0.06	3.01	0.07	3.20	0.04	3.68	0.09	3.70	0.10
Ho	1.14	0.08	1.18	0.09	1.03	0.06	1.00	0.03	0.88	0.05	0.99	0.05	0.58	0.03	0.64	0.01	0.71	0.04	0.71	0.02
Er	2.82	0.18	2.93	0.30	2.50	0.05	2.45	0.07	2.17	0.05	2.04	0.04	1.42	0.03	1.58	0.02	1.75	0.04	1.74	0.06
Tm	0.30	0.02	0.31	0.03	0.26	0.01	0.26	0.01	0.25	0.01	0.29	0.06	0.15	0.01	0.13	0.02	0.18	0.01	0.18	0.01
Yb	1.32	0.08	1.38	0.13	1.14	0.05	1.14	0.03	1.02	0.05	1.33	0.02	0.67	0.03	0.55	0.01	0.82	0.04	0.82	0.02
Lu	0.16	0.01	0.15	0.03	0.14	0.01	0.15	0.01	0.16	0.01	0.18	0.01	0.081	0.004	0.087	0.001	0.10	0.005	0.101	0.003
∑REE+Y	96.18	1.3	96.62	2.01	91.03	1.57	90.58	1.79	81.52	1.17	80.67	1.21	49.81	0.69	49.61	0.45	61.27	1.14	61.23	1.37

^1^ Results are given as confidence intervals (95%, n ≥ 3).

**Table 3:** Operating parameters for the utilized LA-ICPMS ^1^.

ICP-MS		Laser Ablation	
MS type	Agilent 7700x	Laser type	ArF excimer
RF power, W	1450	Wavelength, nm	193
Plasma gas, L/min Ar	15.0	Pulse duration, ns	5
Auxiliary gas, L/min Ar	1.0	Repetition rate, Hz	5
Make-up gas, L/min Ar *	0.8	Fluence, J/cm^2^	2.51
Detector mode	Dual	Spot size, µm	35
Settling time, ms	0.2	Sampling strategy	Single spot
Dwell time, ms *	5.0	Pulses/spot	200
Sweeps/reading *	1	Carrier gas, L/min He *	0.2 Inner cup 0.6 Main volume
Data collection mode	Time-resolved analysis		

^1^ Parameters with star mark (*) are presented as the default values, which were modified during instrumental optimization.

## Data Availability

Data are contained within the article and Supplementary Materials.

## Supplementary Materials

The following supporting information can be downloaded at: https://www.mdpi.com/article/10.3390/molecules29010051/s1, Figure S1: Mass fraction ratios from LA-ICP-MS and EPMA analyses of all samples analyzed. The LA-ICP-MS analysis was done using 5 Hz of laser repetition, 35 μm of spot size and 2.51 J/cm^2^. Figure S2: Concentration ratios from LA-ICP-MS and SN-ICP-MS analyses of all samples analyzed. The LA-ICP-MS analysis was done using 5 Hz of laser repetition, 35 μm of spot size and 2.51 J/cm^2^. Table S1: Element compositions of scheelite samples from different W-associated deposits.
