# Supplementary material for: A Non-Matrix-Matched Calibration Method for In Situ Major and Trace Element Analysis of Scheelite by Nanosecond LA-ICP-MS
Source: Molecules. 2023 Dec 20;29(1):51. doi: 10.3390/molecules29010051 (PMC10779717; doi:10.3390/molecules29010051)
Supplement: Supplementary file 1 [file molecules-29-00051-s001.zip › molecules-2768711-supplementary.pdf]

molecules

MDPI

Article

A non-matrix-matched calibration method for in situ major and trace element analysis of scheelite by nanosecond LA-ICP-MS

Xijuan Tan [1,*], Honghao Tian [1], Lin Lu [2], Dongyang Xiong [1] and Ting Liang [1]

1 Laboratory of Mineralization and Dynamics, College of Earth Sciences and Land Resources, Chang'an University, 126 Yanta Road, Xi'an, 710054, China; honghao1221@163.com (H.T.); xdyran08@163.com (D.X.); liangt@chd.edu.cn (T.L.)

2 Shaanxi Mineral Resources and Geological Survey, Shaanxi Institute of Geological Survey, Xi'an 710068, China; lulin995280@126.com (L.L.)

* Correspondence: tanxijuan@hotmail.com or tanxijuan@chd.edu.cn; Tel.: +86 029 82339067

Supporting information

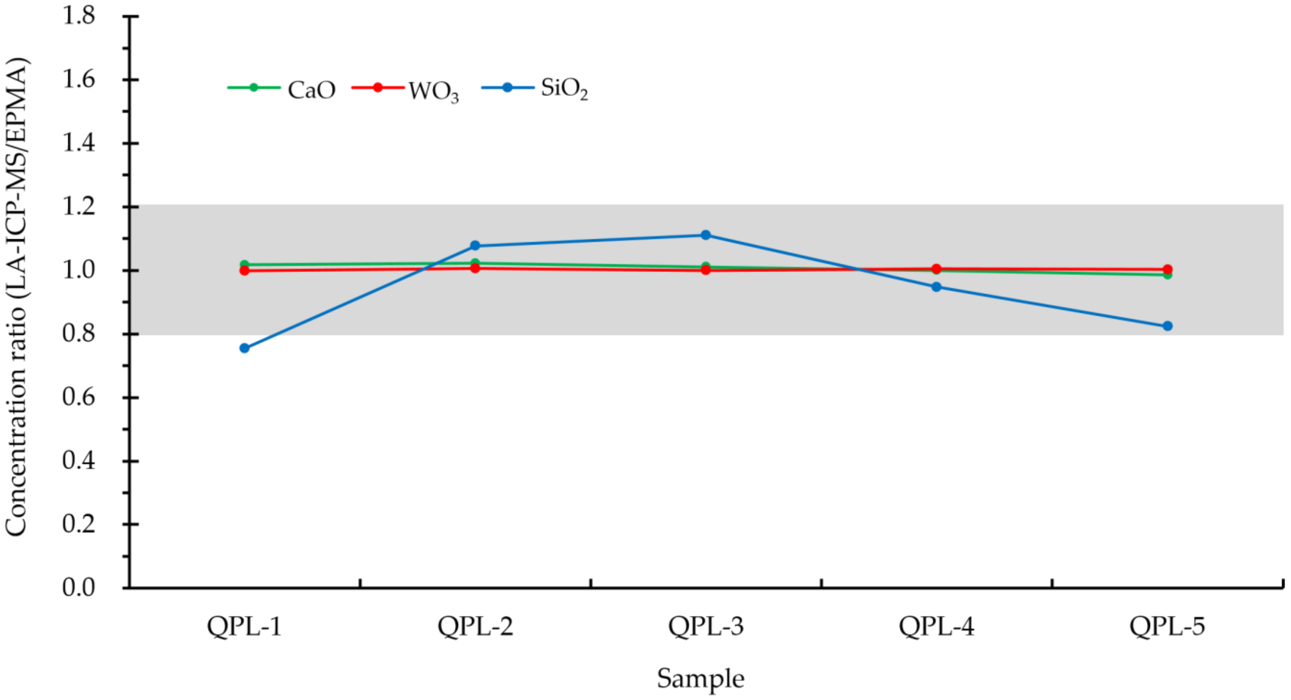

Figure S1. Mass fraction ratios from LA-ICP-MS and EPMA analyses of all samples analyzed. The LA-ICP-MS analysis was done using 5 Hz of laser repetition, 35 µm of spot size and 2.51 J/cm².

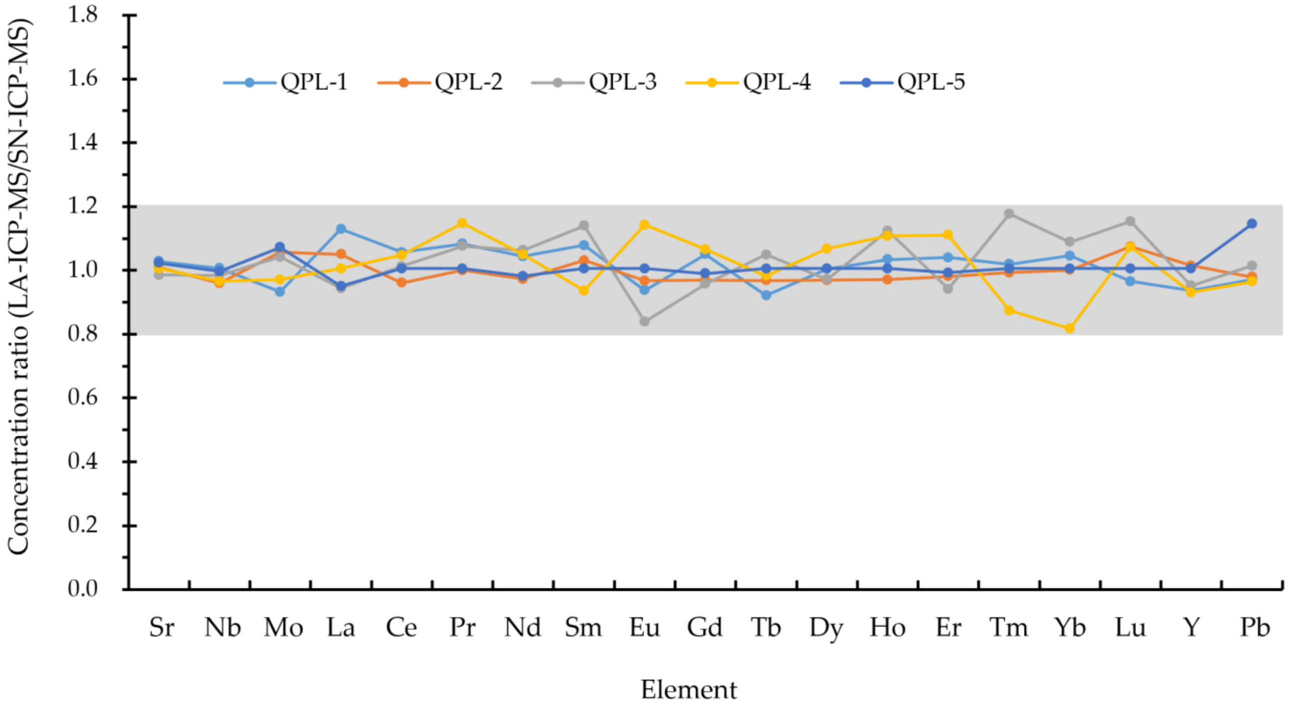

Figure S2. Concentration ratios from LA-ICP-MS and SN-ICP-MS analyses of all samples analyzed. The LA-ICP-MS analysis was done using 5 Hz of laser repetition, 35 µm of spot size and 2.51 J/cm^2.

Table S1. Element compositions of scheelite samples from different W-associated deposits.

Element [1]	CaO	WO_3	Mg	Si	Fe	Sr	Nb	Mo	Pb
QPL-6	19.89	79.82	4.05	815.3	202.6	590.2	4.39	47.15	5.26
QPL-7	19.65	80.06	5.43	749.6	213.1	604.3	3.97	45.79	4.82
QPL-8	19.69	80.05	4.45	637.6	219.6	609.2	3.90	42.57	4.74
QPL-9	19.08	80.64	5.00	597.3	191.7	832.7	3.53	42.06	5.60
QPL-10	19.54	80.19	2.37	706.3	228.6	622.7	3.99	40.82	5.32
Average	19.57	80.15	4.26	701.2	211.1	651.8	3.96	43.68	5.15
Max	19.89	80.64	5.43	815.3	228.6	832.7	4.39	47.15	5.60
Min	19.08	79.82	2.37	597.3	191.7	590.2	3.53	40.82	4.74

Element	CaO	WO_3	Mg	Mn	Fe	Sr	Nb	Mo	Pb
WTG-1	19.13	80.48	17.30	7.79	107.1	61.91	8.53	924.6	2.03
WTG-2	19.38	80.49	28.48	13.48	91.15	23.26	13.56	725.9	2.17
WTG-3	19.89	79.98	43.01	17.19	104.7	21.24	16.60	633.9	2.45
WTG-4	19.02	80.85	42.85	13.06	124.8	19.73	23.75	571.1	2.18
WTG-5	18.79	80.95	14.61	6.27	90.28	39.44	11.81	290.7	2.79
WTG-6	19.58	80.35	20.24	7.16	95.08	41.28	6.31	259.7	11.21
WTG-7	18.91	80.83	61.32	3.03	91.10	34.36	14.52	17.08	0.41
WTG-8	19.28	80.59	23.18	15.92	68.49	22.74	26.62	645.0	2.18
WTG-9	19.43	80.42	36.17	17.75	70.43	18.31	29.75	673.8	2.21
WTG-10	19.42	80.19	15.34	10.37	96.29	60.81	10.07	878.2	1.94
Average	19.28	80.51	30.25	11.20	93.94	34.31	16.15	562.0	2.96
Max	19.89	80.95	61.32	17.75	124.8	61.91	29.75	924.6	11.21
Min	18.79	79.98	14.61	3.03	68.49	18.31	6.31	17.08	0.41

Element	CaO	WO_3	Na	Mg	Mn	Fe	Ga	Sr	Nb	Mo	Pb	Th	U
XDS-1	18.82	80.59	64.98	63.01	72.87	75.15	2.08	351.2	33.20	0.69	19.95	0.34	0.16
XDS-2	19.70	79.91	41.42	63.70	70.26	71.21	3.06	348.4	44.68	0.78	19.49	0.50	0.08
XDS-3	18.97	80.73	54.64	58.93	69.19	79.51	1.94	379.5	30.47	0.77	21.22	0.27	0.05
XDS-4	18.86	80.57	63.96	64.92	72.04	119.7	2.29	338.3	41.89	0.68	22.64	0.19	0.12
XDS-5	19.28	80.38	58.34	59.66	93.08	70.04	2.32	318.4	63.24	0.37	22.10	0.46	0.20
XDS-6	19.09	80.57	67.54	65.23	97.85	78.43	1.65	287.4	70.68	0.49	23.33	0.27	0.23
XDS-7	19.25	80.24	90.70	75.52	85.85	99.91	1.76	305.8	62.37	1.02	20.57	0.35	0.20
XDS-8	19.88	79.76	95.55	63.25	90.16	69.85	2.45	297.0	70.22	0.23	21.72	0.36	0.25
XDS-9	19.08	80.57	81.09	62.02	97.33	78.68	1.27	295.1	67.16	0.43	25.39	0.44	0.19
XDS-10	18.64	80.88	71.82	62.00	92.72	79.38	1.65	301.9	60.76	0.79	26.15	0.38	0.19
Average	19.16	80.42	69.00	63.82	84.13	82.19	2.05	322.3	54.47	0.63	22.26	0.36	0.17
Max	19.88	80.88	95.55	75.52	97.85	119.7	3.06	379.5	70.68	1.02	26.15	0.50	0.25
Min	18.64	79.76	41.42	58.93	69.19	69.85	1.27	287.4	30.47	0.23	19.49	0.19	0.05

Element	Y	La	Ce	Pr	Nd	Sm	Eu	Gd
QPL-6	32.63	3.51	14.04	2.76	16.50	5.66	2.39	6.83
QPL-7	30.99	3.60	14.67	2.81	16.07	4.88	2.25	7.23
QPL-8	29.49	3.65	15.56	2.76	16.28	5.22	2.15	6.37
QPL-9	25.12	3.50	16.80	3.33	19.30	6.37	3.89	8.57

QPL-10	31.56	3.07	13.61	2.82	15.61	5.97	3.12	8.43
Average	29.96	3.46	14.94	2.90	16.75	5.62	2.76	7.49
Max	32.63	3.65	16.80	3.33	19.30	6.37	3.89	8.57
Min	25.12	3.07	13.61	2.76	15.61	4.88	2.15	6.37
Element	Y	La	Ce	Pr	Nd	Sm	Eu	Gd
WTG-1	3.05	2.20	6.10	0.76	2.27	0.95	LD	0.73
WTG-2	23.33	2.08	8.98	2.05	8.24	4.49	2.70	6.64
WTG-3	28.79	2.65	12.72	2.47	14.61	7.89	3.37	10.84
WTG-4	24.75	2.97	16.08	4.22	28.38	10.56	4.31	15.99
WTG-5	6.68	0.82	3.67	0.60	3.34	1.41	0.33	1.79
WTG-6	5.23	0.37	1.38	0.14	0.82	LD	0.17	0.57
WTG-7	0.86	0.03	0.25	0.05	DL	LD	0.09	0.20
WTG-8	31.82	4.52	14.79	2.91	14.22	4.85	2.60	8.72
WTG-9	33.15	14.98	52.57	9.77	56.72	17.40	5.68	19.81
WTG-10	6.17	2.18	8.57	1.28	5.53	1.41	0.21	1.80
Average	16.38	3.28	12.51	2.42	14.90	6.12	2.16	6.71
Max	33.15	14.98	52.57	9.77	56.72	17.40	5.68	19.81
Min	0.86	0.03	0.25	0.05	0.82	LD	LD	0.20
Element	Y	La	Ce	Pr	Nd	Sm	Eu	Gd
XDS-1	119.5	166.4	588.0	94.69	461.1	121.0	27.95	122.5
XDS-2	185.4	165.4	685.7	128.2	625.4	173.1	38.97	162.0
XDS-3	99.33	176.9	583.4	90.36	428.3	109.4	19.52	100.9
XDS-4	154.5	122.8	476.0	83.10	420.3	135.2	30.12	138.0
XDS-5	235.8	130.4	500.3	87.33	463.3	149.5	67.52	180.2
XDS-6	244.9	142.4	528.6	93.06	411.8	135.5	85.16	170.7
XDS-7	220.8	119.1	461.6	81.33	432.2	148.6	67.45	176.2
XDS-8	260.1	131.6	506.7	86.61	424.7	140.4	80.84	170.3
XDS-9	266.1	149.4	534.5	86.98	412.3	136.3	80.43	169.4
XDS-10	227.0	102.3	429.9	75.03	362.3	133.5	79.55	147.2
Average	201.3	140.7	529.5	90.67	444.2	138.3	57.75	153.8
Max	266.1	176.9	685.7	128.2	625.4	173.1	85.16	180.2
Min	99.33	102.3	429.9	75.03	362.3	109.4	19.52	100.9
Element	Tb	Dy	Ho	Er	Tm	Yb	Lu	∑REE+Y
QPL-6	0.89	6.00	1.21	3.23	0.33	1.31	0.18	97.47
QPL-7	1.05	5.52	1.18	2.78	0.32	1.41	0.17	94.93
QPL-8	0.97	5.44	1.01	2.98	0.23	1.06	0.13	93.31
QPL-9	1.21	5.87	1.10	2.35	0.22	0.74	0.04	98.42
QPL-10	1.22	6.81	1.24	2.82	0.32	1.32	0.15	98.07
Average	1.07	5.93	1.15	2.83	0.28	1.17	0.13	96.44
Max	1.22	6.81	1.24	3.23	0.33	1.41	0.18	98.42
Min	0.89	5.44	1.01	2.35	0.22	0.74	0.04	93.31
Element	Tb	Dy	Ho	Er	Tm	Yb	Lu	∑REE+Y
WTG_1	0.16	0.80	0.14	0.28	0.06	0.38	LD	17.88

WTG_2	1.15	7.83	1.28	3.60	0.33	1.63	0.13	74.46
WTG_3	1.63	10.14	1.82	4.41	0.33	1.61	0.23	103.5
WTG_4	2.20	12.16	1.73	4.00	0.34	0.92	0.13	128.7
WTG_5	0.36	1.54	0.32	0.87	0.09	0.44	0.10	22.36
WTG_6	0.12	0.64	0.17	0.81	0.08	0.51	0.07	11.08
WTG_7	LD	0.15	0.06	0.11	0.00	0.03	0.00	1.82
WTG_8	1.54	10.21	1.80	4.03	0.48	2.19	0.14	104.8
WTG_9	3.11	15.81	2.68	5.05	0.38	1.34	0.22	238.7
WTG_10	0.24	2.03	0.39	0.82	0.10	0.41	0.03	31.16
Average	1.17	6.13	1.04	2.40	0.22	0.95	0.12	73.45
Max	3.11	15.81	2.68	5.05	0.48	2.19	0.23	238.7
Min	LD	0.15	0.06	0.11	0.00	0.03	LD	1.82

Element	Tb	Dy	Ho	Er	Tm	Yb	Lu	∑REE+Y
XDS_1	19.49	118.6	21.14	47.97	5.43	23.15	2.20	1939
XDS_2	26.46	156.7	26.50	62.39	5.90	28.53	2.61	2473
XDS_3	16.13	102.7	17.97	41.38	4.45	20.44	1.79	1813
XDS_4	23.15	140.7	25.62	55.98	5.65	24.91	2.18	1838
XDS_5	29.51	185.4	30.62	65.70	6.16	23.29	2.25	2157
XDS_6	29.27	175.4	28.89	58.96	5.81	25.73	1.93	2138
XDS_7	29.71	173.7	28.70	60.77	5.58	25.43	2.20	2033
XDS_8	31.72	183.7	29.82	66.40	5.84	25.64	2.16	2147
XDS_9	30.39	184.5	30.55	66.65	5.97	25.59	2.21	2181
XDS_10	28.66	175.1	27.57	59.90	5.57	21.12	1.87	1877
Average	26.45	159.6	26.74	58.61	5.64	24.38	2.14	2060
Max	31.72	185.4	30.62	66.65	6.16	28.53	2.61	2473
Min	16.13	102.7	17.97	41.38	4.45	20.44	1.79	1813

[1] Matrix CaO and WO_3 were shown in wt. %, and trace elements were given in μg/g. All the results were given in average value, and the determination RSDs were less than 5% ($n \geq 3$).
